# Supplementary material for: Effect of advanced periodontal self-care in patients with early-stage periodontal diseases on endothelial function: An open-label, randomized controlled trial
Source: PLoS One. 2021 Sep 23;16(9):e0257247. doi: 10.1371/journal.pone.0257247 (PMC8459983; doi:10.1371/journal.pone.0257247)
Supplement: S1 File — (DOCX) [file pone.0257247.s002.docx]

**Effect of advanced periodontal self-care in patients with early stage periodontal diseases on endothelial function: A randomized controlled trial**

**Clinical Protocol**

**Ver. 1.2 (Final ver.)**

**30 June 2017**

**Principal Investigator:**

Nobuhiro Hanada, DDS, PhD

Department of Translational Research,

Tsurumi University School of Dental Medicine, Yokohama, Japan

TEL: 045-580-8462

FAX: 045-573-2473

E-mail: hanada-n@tsurumi-u.ac.jp

**Funded by:**

Japan Society for the Promotion of Science

SECOM Science and Technology Foundation

PROTOCOL SUMMARY

| Title | Effect of advanced periodontal self-care in patients with early stage periodontal diseases on endothelial function: A randomized controlled trial |
| --- | --- |
| Study Description | To evaluate the effect of periodontal self-care in patients with early stage periodontal diseases on endothelial function, the changes of ACVD-related vascular function markers FMD and serum ADMA level were compared between standard care group and advanced care group. |
| Study Design | Parallel group, 3-month follow-up, open-label, randomized controlled trial. |
| Study Duration | Estimated duration for the main protocol (from start of screening to last subject processed and finishing the study) is approximately 2.5 years |
| Study Center | Department of Translational Research, Tsurumi University School of Dental Medicine, Yokohama, Japan |
| Objectives | Primary Objective: To evaluate the efficacy of advanced periodontal self-care on endothelial function  Secondary Objectives: To evaluate the association between FMD and serum ADMA level |
| Number of Subjects | 110 randomized patients in two arms; advanced self-care in addition to standard care and standard care |
| Inclusion/Exclusion Criteria | Inclusion Criteria   - Male and female, 20-70 years old, in any distribution - Having ≥20 functioning teeth. - Diagnosis of early stage periodontal diseases. - Consent and compliance with all aspects of the study protocol, methods, providing data during follow-up contact.   Exclusion Criteria   - Difficulty in traveling alone to the institution. - Diagnosis of severe periodontal diseases. - Necessity for prosthodontic treatment. - Presence of an untreated carious cavity. - Presence of a partially impacted tooth. - having used systemic antibiotics within the previous 3 months. - Used regular medication for any other chronic disease. - Involvement with any other ongoing studies. |
| Intervention | Test Group: Will receive disinfectant application using custom-fabricated tray in addition to standard care for periodontal diseases  Control Group: Will receive standard care for periodontal diseases |

PROTOCOL SCHEMA


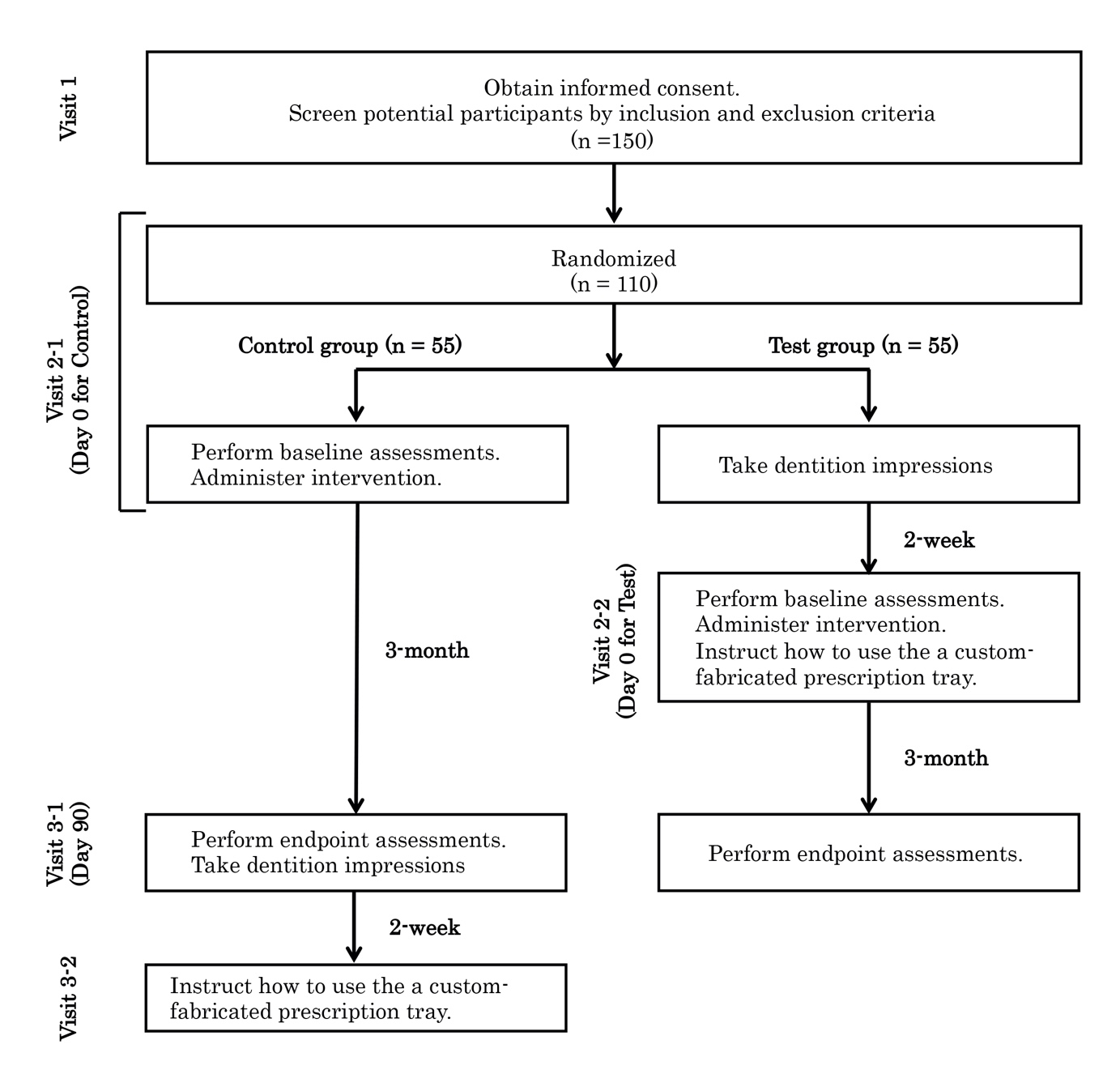


SCHEDULE OF ACTIVITIES

|  | **Visit 1** | **Visit 2-1** | **Visit 2-2** | **Visit 3-1** | **Visit 3-2** |
| --- | --- | --- | --- | --- | --- |
| **Procedures** | Screening  Day -60 to -1 | Randomization  Baseline assessment  Day 0 (Control) | Baseline assessment  Day 0 (Test) | Final assessment  Day 90 ± 3 | Custom tray setting for control  (Optional) |
| Informed consent | X |  |  |  |  |
| Demographics | X |  |  |  |  |
| Dental history | X |  |  |  |  |
| Medical history | X |  |  |  |  |
| Concomitant medication review | X | X | X | X | X |
| Randomization | X |  |  |  |  |
| Dental examination ^a^ | X | X | X | X | X |
| FMD measurement |  | X | X | X |  |
| Serum chemistry ^b^ |  | X | X | X |  |
| Blood pressure measurement |  | X | X | X |  |
| Dentition impressions |  | X |  | X |  |
| Administer study intervention |  | X | X |  |  |
| Adverse event review and evaluation | X | X | X | X | X |
| ^a^ bleeding on probing, periodontal pocket depth; ^b^ ADMA, C-reactive protein | | | | | |

**Purpose**

The primary objective is to evaluate the efficacy of advanced periodontal self-care in patients with early stage periodontal diseases on endothelial function.

**Background**

A remarkable association between periodontal disease and atherosclerotic cardiovascular disease (ACVD) has been reported, although their causative relationship remains controversial.

**Study Rationale**

Entry of oral bacteria, including periodontal pathogens and/or their products into the bloodstream, is common regardless of periodontal status. The immune response following persistent bacteremia from periodontal lesions may lead to ACVD. Identification of periodontal care effectiveness against ACVD require well-designed interventional studies.

Endothelial dysfunction occurs in the early stages of atherosclerosis and can be assessed by measuring flow-mediated dilatation (FMD) of the brachial artery. Therefore, FMD appears to be an appropriate surrogate marker to evaluate periodontal care effectiveness against ACVD.

**Goals of the Study**

1. To evaluate the efficacy of advanced periodontal self-care on endothelial function as measured by FMD and serum asymmetric dimethylarginine (ADMA; an endogenous NO synthase inhibitor) level compared to the control group.

2. To evaluate the association between FMD and serum ADMA level.

**Duration of the Study**

The study is estimated to complete enrollment within 6 months from study initiation. The duration of this study for each subject will be a maximum of two and a half years.

**Advanced Periodontal Self-care:**

Advanced periodontal self-care involves application of disinfectant with a custom-fabricated prescription tray to the teeth surface and periodontal pockets in addition to standard periodontal care.

**Product Intended Use**

Advanced periodontal self-care is to be used for a removal of periodontal and cariogenic pathogen from oral cavity.

**Product Acquisition**

The disinfectant and tray will be purchased by Department of Translational Research, Tsurumi University School of Dental Medicine from Medoc International Co. Ltd. and Shiken Corp., respectively at no cost to the patient.

**Potential Risks and Benefits to Patients**

Every effort will be taken to prevent medical accidents. There are no potential risks to patients due to a patient being treated with standard care and/or advanced periodontal self-care.

Because each subject will be received standard of care for periodontal diseases, improvement of periodontal health condition will be expected. Improvement of endothelial function will also be expected if our theory is correct.

**Methods**

*Study Design*.

The study will be designed as a parallel group (1:1), 3-month follow-up, open-label, randomized controlled trial.

One control group of 55 subjects will undergo a typical standard care for periodontal diseases. The other test group of 55 will undergo an application of hypochlorous acid water with a custom-fabricated prescription tray in addition to standard care.

*Study Population and Selection Criteria*.

All aspects of the study and consent forms will be Institutional Review Board (IRB) approved prior to implementation.

All participants will require full informed consent, be willing and able to comply with all study requirements and will meet the following criteria;

- Male or female aged 18-70
- 20 or more functioning teeth
- Periodontitis with bleeding on probing (more than two sites) and/or with 4 mm or larger periodontal pocket depth (more than one site)

Subjects will be excluded from the study based on the following criteria;

- Difficulty in traveling alone to the institution
- Necessity for prosthodontic treatment
- Presence of an untreated carious cavity
- Having used systemic antibiotics within the previous 3 months
- Used regular medication for any other chronic disease
- Any other treatment in higher-priority than periodontal treatment
- Involvement with any other ongoing studies

*Recruitment methods*.

Subjects will be recruited from advertisements in social networking services, Tsurumi University Dental Hospital in Yokohama City, Ariyoshi Dental Clinic in Tokyo, and Kioicho Plaza Clinic in Tokyo.

*Data collection and reporting*.

Data will be collected at the baseline and 3 months after intervention at Tsurumi University Dental Hospital, Ariyoshi Dental Clinic or Kioicho Plaza Clinic. Data from the study will be maintained in Department of Translational Research, Tsurumi University School of Dental Medicine for 5 years after the date the investigation is published.

*Expected outcomes*.

Both standard care for periodontal diseases and advanced periodontal care will show improved patient outcomes on periodontal diseases.

It is the expectation that application of disinfectant to periodontal tissue with a custom-fabricated prescription tray will show improved patient outcomes on endothelial function (FMD and serum ADMA level) over standard care for periodontal diseases.

*Adverse reactions*.

There is no expectation of any adverse outcomes or reactions due to a patient being treated with standard care and/or advanced periodontal self-care.

All participants will be given access to contact info of the principal investigator or co-investigators. Any adverse reactions should be reported immediately to the principal investigator or co-investigators.

**Reasons for Withdrawal or Termination**

A subject may be discontinued from the study at any time if the subject or the investigators feels that it is not in the subject’s best interest to continue. The following is a list of possible reasons for study treatment discontinuation:

- Screening Failure
- Subject withdrawal of consent
- Subject is not compliant with study procedures
- Adverse Event that in the opinion of the Investigator would be in the best interest of the subject to discontinue study participation
- Protocol violation requiring discontinuation
- Lost to follow-up
- Contract diseases that make it difficult to continue research
- Subject death

All subjects are free to withdraw from participation at any time, for any reason, specified or unspecified, and without prejudice. Reasonable attempts will be made by the Investigator to provide a reason for subject withdrawals. The reason for the subject’s withdrawal from the study will be specified in the subject’s source documents and the Case Report Form. If a subject is withdrawn from treatment due to an adverse event), the subject will be followed and treated by the Investigator until the abnormal parameter or symptom has resolved or stabilized. The Investigator must make every effort to contact subjects who are lost to follow-up.

**Handling of Participant Withdrawals of Termination**

Although subjects may withdraw from the study at any time and for any reason, (or may be withdrawn at the Investigator’s discretion), subject withdrawal should be avoided as much as reasonably possible. In any case, appropriate follow-up for endpoints should be continued. Subjects who prematurely discontinue are not to be replaced. For subjects considered lost to follow-up, the Case Report Form must be completed up to the last visit performed.

**Premature Termination or Suspension of Study**

This study may be temporarily suspended or prematurely terminated if there is sufficient reasonable cause. Written notification, documenting the reason for study suspension or termination, will be provided by the suspending or terminating party to the investigators and the IRB, as appropriate. If the study is prematurely terminated or suspended, the principal investigator will promptly inform the IRB and will provide the reason(s) for the termination or suspension. Circumstances that may warrant termination or suspension include, but are not limited to:

- Determination of unexpected, significant, or unacceptable risk to participants
- Demonstration of efficacy that would warrant stopping
- Insufficient compliance to protocol requirements
- Data that are not sufficiently complete and/or evaluable
- Determination of futility

Study may resume once concerns about safety, protocol compliance and data quality are addressed and satisfy the IRB.

**Methods and Study Schedule**

Subjects eligible for the study will review and undergo informed consent. Once consented, subjects will be randomly assigned on a 1:1 basis to undergo:

- Control Group: Current standard care for periodontal diseases
- Test Group: Application of disinfectant with a custom-fabricated prescription tray in addition to standard care

*Visit for Screening (-60 to -1 days from Day 0)*

The following procedures will be performed at the Screening visit:

- Review the study with the subject and obtain written informed consent
- Assign the subject a unique screening/enrollment number
- Record demographics (age, gender)
- Review and record medical history, surgical history, and medication history to determine eligibility based on inclusion/exclusion criteria
- Document all current medications, including medications over-the-counter and herbal medications

*Visit for Treatment (Day 0)*

After baseline measurements, the following techniques will be used according to the arm the patient is randomized to:

Control Group:

The 55 subjects will undergo typical standard care for periodontal diseases.

Treatment Group:

The 55 subjects will undergo application of disinfectant with a custom-fabricated prescription tray in addition to standard care.

*Visit for endpoint measurements (Day 90)*

The following procedures will be performed at the visit for endpoint measurements:

- Assess for adverse events
- Document all current medications, including medications over-the-counter and herbal medications

*Unscheduled Visit*

The following procedures will be performed if the subject presents to the clinic at any other time point not specified above:

- Assess for adverse events
- Document all current medications, including medications over-the-counter and herbal medications

*Early Termination*

All subjects have the right to withdraw from study participation at any time during the study. If, for whatever reason, a subject withdraws from the study, an Early Termination visit will be performed.

The following procedures will be performed (if agreed upon by the subject) at the Early Termination visit:

- Assess for adverse events
- Document all current medications, including medications over-the-counter and herbal medications

**Randomization**

The eligible patients will be allocated randomly to the test or control group via the envelope method stratified by the smoking habit, which is a common risk factor for ACVD and periodontal diseases.

**Sample Size Calculations**

The sample size was calculated based on a previous study that a sample size of 90 was needed to detect a 1% difference in FMD (primary outcome) between the two groups, with a standard deviation of the mean difference of 1.67% at a 2-sided α error of 0.05 and 80% power.

**Statistical Analysis Plan**

Primary Endpoint: FMD.

Secondary Endpoint: Serum ADMA level.

All analyses will be performed using per-protocol population as well as intention-to-treat population.

**Assessment of Safety**

Adverse events will be monitored and collected by the study team from the point of signed consent until the last day of study participation. For each adverse event, a detailed explanation will be obtained from the subject and subject’s medical record. All adverse event will be recorded on the Case Report Form.

*Definition of Adverse Event*.

An adverse event is defined as any unanticipated medical occurrence regardless to relationship of the investigative arm of the trial. An adverse event can be any unintended sign, lab abnormality, symptom, or disease associated with the trial. Any abnormality that presents during a medical test are to be defined as an adverse event if it produces clinical signs and/or symptoms, requires intervention, or deemed clinically significant by the Principal Investigator

**Data Handling and Record Keeping**

The collection of personal patient information will be limited to the amount necessary to achieve the aims of the research, so that no unneeded sensitive information is being collected.

Only study personnel will collect data. Hard copy documents will be retained for the duration of the study until data entry. All hard copy documents will be kept in a locked cabinet in the Department office. Data entry will be completed in the isolated secure persomal computer. Data will be exported into Excel file format (password protected), which will then be used for data analysis. Only de-identified data will be used for data analysis. All hard copy documents will be shredded within five years after publication.

**Institutional Review Board**

The protocol, informed consent form(s), and all participant materials will be submitted to the IRB for review and approval. Approval of both the protocol and the consent form must be obtained before any participant is enrolled. Any amendment to the protocol will require review and approval by the IRB before the changes are implemented to the study. All changes to the consent form will be IRB approved; a determination will be made regarding whether previously consented participants need to be re-consented.

**Protocol Deviation**

A protocol deviation is any noncompliance with the clinical trial protocol. The noncompliance may be either on the part of the participant, the investigators, or the study site staff. As a result of deviations, corrective actions are to be developed by the site and implemented promptly.

All protocol deviations/violations should be documented using the Protocol Deviations/Violations Case Report Form and submitted to the IRB according to their reporting guidelines.

**Consent Process**

Each potential subject must provide written consent with full knowledge of the procedures involved. The informed consent, approved by the IRB and in accordance with regulatory guidelines, must be fully explained by the Investigator or member of the study staff including the study aims, methods, benefits and risks, and signed by the subject before enrollment into the study. Potential subjects will be informed that study participation is voluntary and that they may withdraw at any time. The subjects will be told that choosing against participation will not affect the care received for treatment. The subject will be given sufficient time to read the consent and ask any questions. Once the informed consent is signed, the subject will be given a copy of the document.

**Laws and Regulations**

This clinical study will be conducted in compliance with national laws and regulations, as well as any applicable guidelines. The trial will be registered on UMIN Clinical Trials Registry ([www.umin.ac.jp/ctr/](http://www.umin.ac.jp/ctr/)).

**Publication and Data Sharing Policy**

The publication of manuscripts containing the study results shall be in accordance with a process determined by the co-investigators.

**Study Personnel and Roles**

| Nobuhiro Hanada | Principal Investigator | Responsible for all study related issues |
| --- | --- | --- |
| Meu, Ariyoshi | Co-Project Manager | Data management and data collection |
| Ayako Okada | Co-Project Manager | Addresses IRB issues and data collection |
| Takatoshi Murat | Data Manager | Statistical analysis |
| Khairul Matin | Co-Investigators | Data collection |
| Ryoko Otsuka | Co-Investigators | Data collection |
| Mamiko Yamashita | Co-Investigators | Data collection |
| Masayuki Suzuki | Co-Investigators | Data collection |
| Rumi Wakiyama | Co-Investigators | Data collection |
| Ken Tateno | Co-Investigators | Data collection |
| Kanako Saji | Co-Investigators | Data collection |
| Hiroshi Kawahara | Co-Investigators | Data collection |
| Megumi Suzuki | Co-Investigators | Standard care for periodontal diseases |
| Hitomi Aoyagi | Co-Investigators | Standard care for periodontal diseases |
| Hiromi Uematsu | Co-Investigators | Standard care for periodontal diseases |
| Akiko Imamura | Co-Investigators | Standard care for periodontal diseases |
| Miki Kosaka | Co-Investigators | Data collection |
| Tomoko Mizukaki | Co-Investigators | Data collection |
| Tsutomu Sato | Co-Investigators | Data collection |
| Yoshiaki Nomura | Co-Investigators | Data collection |
| Hidenori Yamada | Co-Investigators | Data collection |
| Mayu Miyanohara | Co-Investigators | Data collection |
| Kaoru Sogabe | Co-Investigators | Data collection |
| Shigeru Kawashima | Co-Investigators | Data collection |
| Kenichi Nebuka | Co-Investigators | Data collection |
| Takashi Ariyoshi | Co-Investigators | Data collection |
| Yuri Hanada | Co-Investigators | Data collection |
| Toshiko Ariyoshi | Co-Investigators | Data collection |
| Iu Shimizu | Co-Investigators | Data collection |
| Maiko Kikuchi | Co-Investigators | Data collection |

**Funding**

This trial will be supported by the Japan Society for the Promotion of Science and the SECOM Science and Technology Foundation. The funders had no role in the study design, data collection and analysis, decision to publish, or preparation of the manuscript.

**Conflicts of Interest**

Khairul Matin and Nobuhiro Hanada received research grant from Medoc International Co. Ltd; Nobuhiro Hanada received a research grant from Shiken Corp. There are no other declared potential conflicts of interest.

**Abbreviations**

| ACVD | Atherosclerotic Cardiovascular Disease |
| --- | --- |
| ADMA | Asymmetric Dimethylarginine |
| FMD | Flow-Mediated Dilatation |
| IRB | Institutional Review Board |

**Protocol Amendment History**

| **Version** | **Date** | **Description of Change** | **Brief Rationale** |
| --- | --- | --- | --- |
| 1.0 | 19/7/2016 | - | - |
| 1.1 | 28/2/2017 | Study Personnel | Added co-investigators for data collection |
| 1.2 | 30/6/2017 | Study Personnel | Added co-investigators for data collection |

**References**

Lockhart PB, Bolger AF, Papapanou PN, et al. Periodontal disease and atherosclerotic vascular disease: Does the evidence support an independent association?: A scientific statement from the American heart association. *Circulation* 2012; 125: 2520–2544.

Olsen I. Update on bacteraemia related to dental procedures. *Transfus Apher Sci* 2008; 39: 173–178.

Kebschull M, Demmer RT, Papapanou PN. ‘Gum bug, leave my heart alone!’-epidemiologic and mechanistic evidence linking periodontal infections and atherosclerosis. *J Dent Res* 2010; 89: 879–902.

Widlansky ME, Gokce N, Keaney JF, et al. The clinical implications of endothelial dysfunction. *J Am Coll Cardiol* 2003; 42: 1149–1160.

Cohn JN, Quyyumi AA, Hollenberg NK, et al. Surrogate markers for cardiovascular disease: Functional markers. *Circulation* 2004; 109: IV31–IV46.

Putt MS, Proskin HM. Custom tray application of peroxide gel as an adjunct to scaling and root planing in the treatment of periodontitis: A randomized, controlled three-month clinical trial. *J Clin Dent* 2012; 23: 48–56.

Tonetti MS, D’Aiuto F, Nibali L. Treatment of periodontitis and endothelial function. *New Engl J Med* 2007; 356: 911–20.

Juonala M, Viikari JSA, Alfthan G, et al. Brachial artery flow-mediated dilation and asymmetrical dimethylarginine in the cardiovascular risk in young Finns study. *Circulation* 2007; 116: 1367–1373.
