# Supplementary figures and images for: Effect of advanced periodontal self-care in patients with early-stage periodontal diseases on endothelial function: An open-label, randomized controlled trial
Source: PLoS One. 2021 Sep 23;16(9):e0257247. doi: 10.1371/journal.pone.0257247 (PMC8459983; doi:10.1371/journal.pone.0257247)

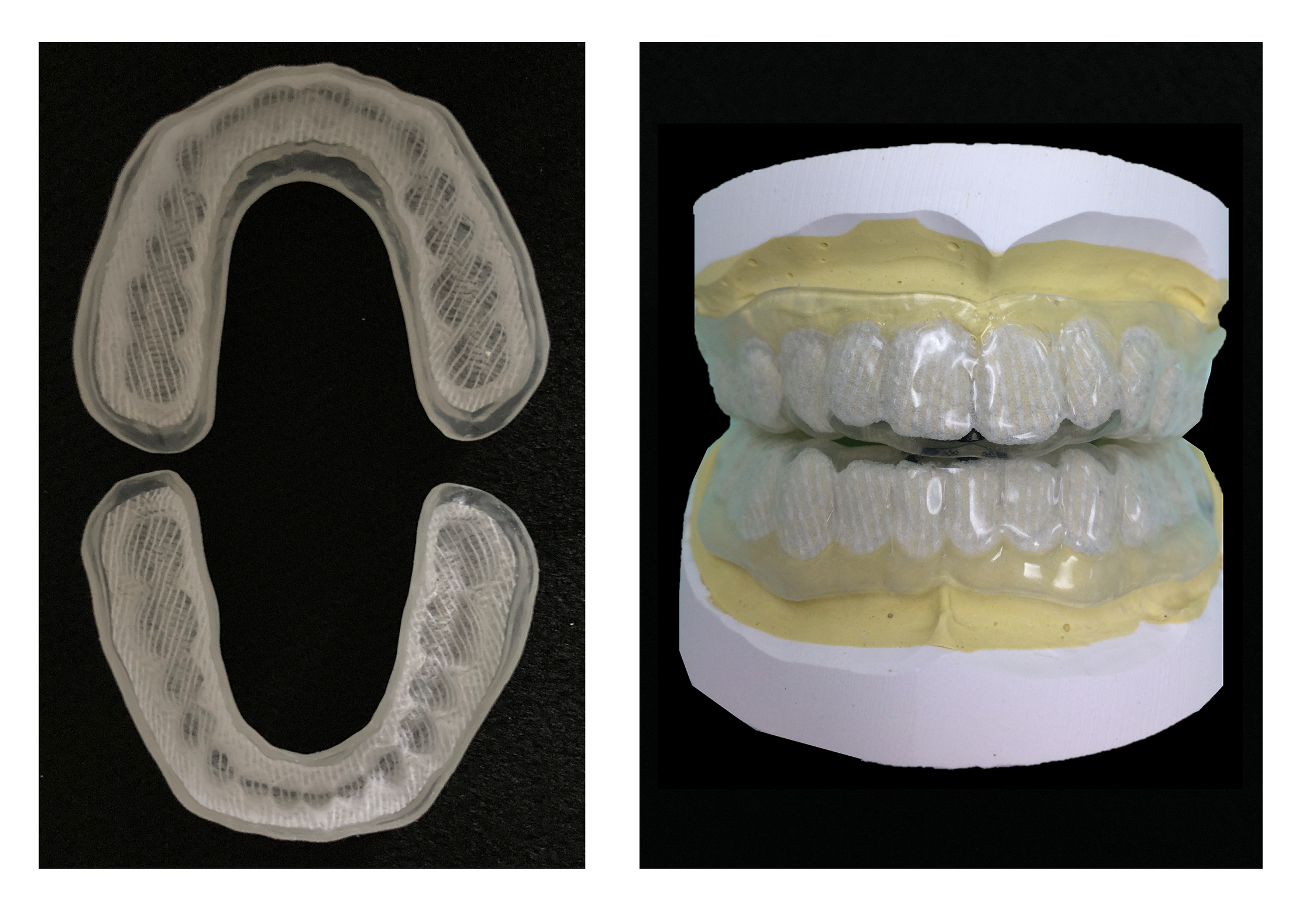

Supplement: S1 Fig — (TIF) [file pone.0257247.s003.tif]

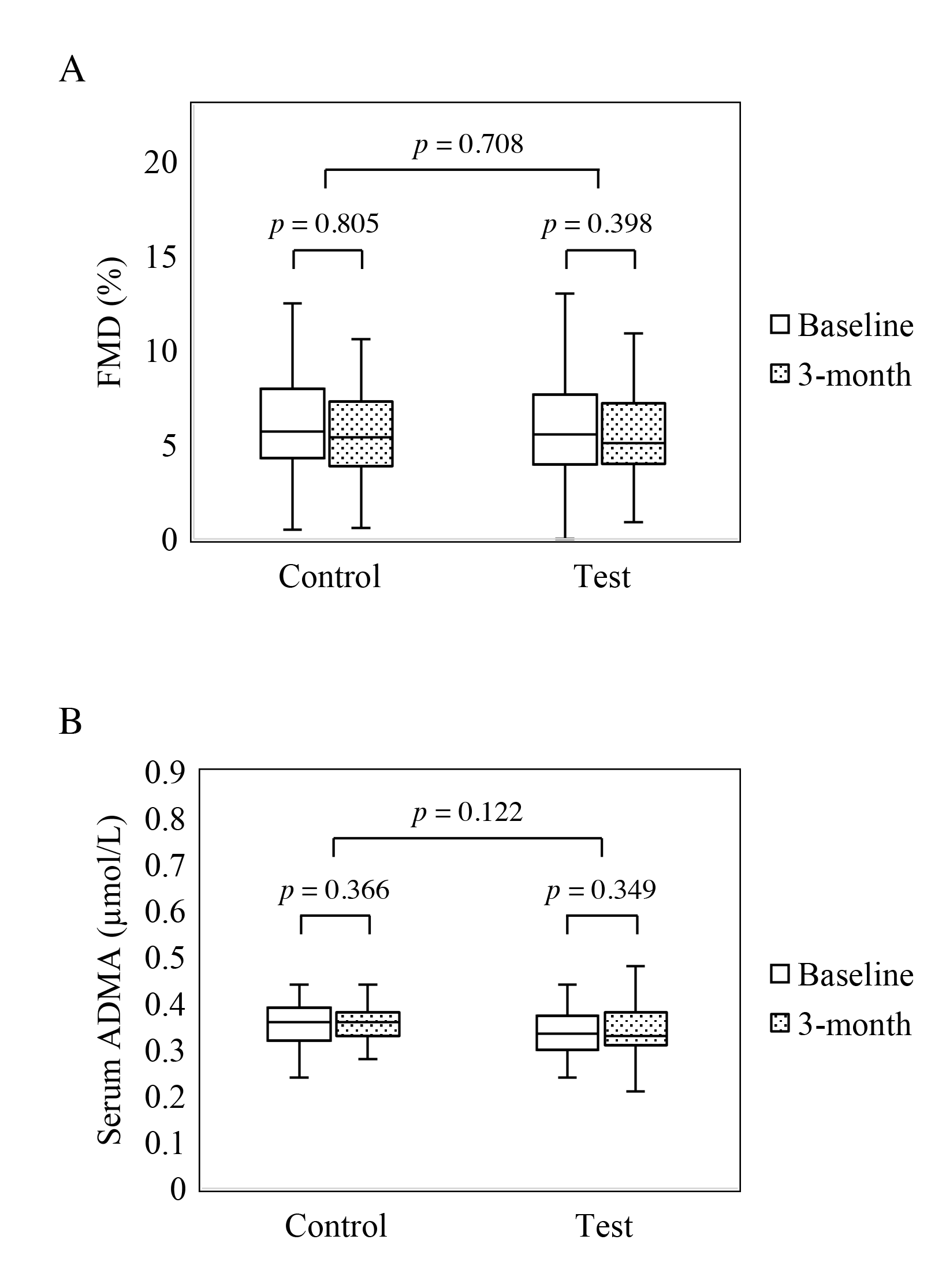

Supplement: S2 Fig — Box and whisker plot of FMD (A) and serum ADMA (B) levels by group. The box contains values between the 25th and 75th percentiles (central line, median). Vertical lines represent the minimum and maximum. P-values were calculated using the paired Student’s t-test (FMD) or Wilcoxon’s signed-rank test (serum ADMA level) for changes from baseline and the unpaired t-test (FMD) or the Mann–Whitney U test (serum ADMA level) for group differences. FMD, brachial artery dilatation; AMDA, asymmetric dimethylarginine. (TIF) [file pone.0257247.s004.tif]

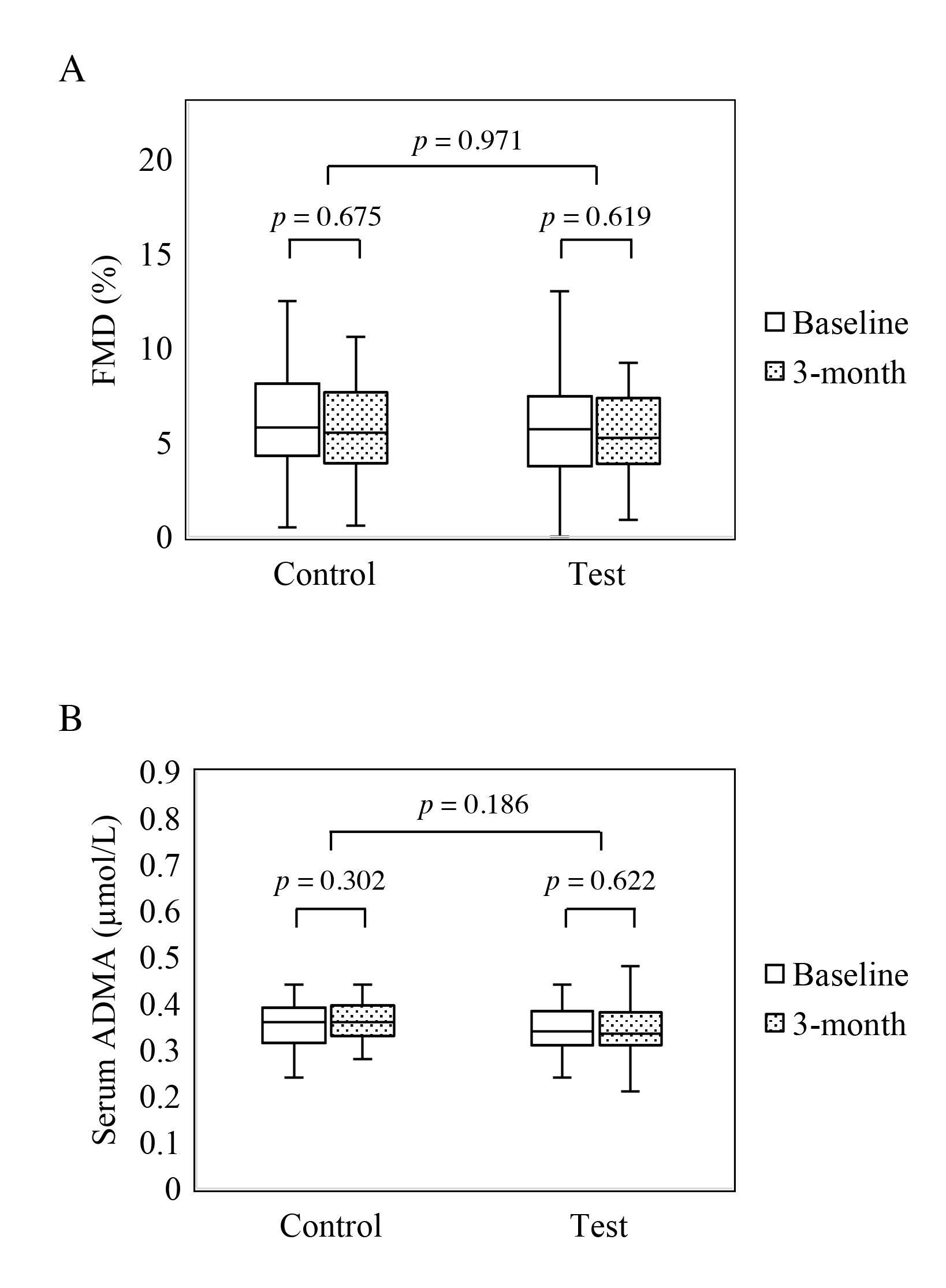

Supplement: S3 Fig — Box and whisker plot of FMD (A) and serum ADMA levels (B) by group. The box contains values between the 25th and 75th percentiles (central line, median) of serum ADMA levels. Vertical lines represent the minimum and maximum. P-values were calculated using the paired Student’s t-test (FMD) or Wilcoxon’s signed-rank test (serum ADMA level) for changes from baseline and the unpaired t-test (FMD) or the Mann–Whitney U test (serum ADMA level) for group differences. FMD, brachial artery dilatation; AMDA, asymmetric dimethylarginine. (TIF) [file pone.0257247.s005.tif]
