# Supplementary material for: Effect of advanced periodontal self-care in patients with early-stage periodontal diseases on endothelial function: An open-label, randomized controlled trial
Source: PLoS One. 2021 Sep 23;16(9):e0257247. doi: 10.1371/journal.pone.0257247 (PMC8459983; doi:10.1371/journal.pone.0257247)
Supplement: S1 Table — (DOCX) [file pone.0257247.s006.docx]

**S1 Table.** Assessment of vascular function in the per-protocol analysis.

|  | Baseline | | |  | | |
| --- | --- | --- | --- | --- | --- | --- |
|  | Control (*n* = 49) | Test (*n* = 42) | |  | | |
| FMD (%), mean ± SD | 6.2 ± 3.1 | 5.7 ± 3.0 | |  | | |
| ADMA (nmol/L), median (IQR) | 0.36 (0.32–0.39) | 0.34 (0.31–0.38) | |  | | |
|  | Endpoint | | | | | |
|  | Control  (*n* = 49) | Test  (*n* = 42) | | Mean difference  (95% CI) | | P value |
| FMD (%), mean ± SD | 5.9 ± 2.5 | 5.5 ± 2.3 | | -0.5 (-1.5–0.5) | | 0.345 |
| ADMA (nmol/L), median (IQR) | 0.36 (0.33–0.40) | 0.34 (0.31–0.38) | | -0.03 (-0.05–-0.00) | | 0.056 |
|  | Improvement | | | | | |
|  | Control (*n* = 49) | | | Test (*n* = 42) | | |
|  | Mean difference  (95% CI) | | P value | Mean difference  (95% CI) | P value | |
| FMD (%) | -0.2 (-1.2 – 0.8) | | 0.675 | -0.2 (-1.2 – 0.7) | 0.619 | |
| ADMA (nmol/L) | 0.01 (-0.00 – 0.02) | | 0.302 | -0.00 (-0.01 – 0.01) | 0.622 | |

P-values were calculated using the unpaired *t*-test (FMD) or the Mann–Whitney U test (serum ADMA level) for group differences at endpoint and the paired Student’s *t*-test (FMD) or Wilcoxon’s signed-rank test (serum ADMA level) for changes from baseline. FMD, flow-mediated dilatation of the brachial artery; ADMA, serum asymmetric dimethylarginine level; SD, standard deviation; IQR, interquartile range; CI, Confidence interval.
