# Supplementary material for: Effect of advanced periodontal self-care in patients with early-stage periodontal diseases on endothelial function: An open-label, randomized controlled trial
Source: PLoS One. 2021 Sep 23;16(9):e0257247. doi: 10.1371/journal.pone.0257247 (PMC8459983; doi:10.1371/journal.pone.0257247)
Supplement: S2 Table — (DOCX) [file pone.0257247.s007.docx]

**S2 Table.** Periodontal status in the per-protocol analysis.

|  | Baseline | | | |  | | |
| --- | --- | --- | --- | --- | --- | --- | --- |
|  | Control (*n* = 49) | Test (*n* = 42) | | |  | | |
| Mean PPD (mm), median (IQR) | 2.1 (1.9–2.3) | 2.0 (1.8–2.2) | | |  | | |
| BOP (%), median (IQR) | 10.1 (5.5–20.0) | 9.3 (4.5–15.5) | | |  | | |
|  | Endpoint | | | | | | |
|  | Control (*n* = 49) | Test (*n* = 42) | | | Mean difference  (95% CI) | | P value |
| Mean PPD (mm), median (IQR) | 1.9 (1.8 – 2.1) | 1.9 (1.8 – 2.0) | | | -0.1 (-0.1 – -0.0) | | 0.088 |
| Mean PPD (mm), median (IQR) | 8.9 (3.7 – 16.4) | 7.9 (2.9 – 14.9) | | | -2.4 (-6.1 – 1.4) | | 0.540 |
|  | Improvement | | | | | | |
|  | Control (*n* = 49) | | | Test (*n* = 42) | | | |
|  | Mean difference  (95% CI) | | P value | Mean difference  (95% CI) | | P value | |
| Mean PPD (mm) | 0.2 (0.1 – 0.2) | | <0.001 | 0.2 (0.1 – 0.2) | | <0.001 | |
| BOP (%) | 1.7 (1.2 – 4.7) | | 0.078 | 1.3 (-0.2 – 2.9) | | 0.085 | |

Mann–Whitney U test was used for comparisons between groups at endpoint. Wilcoxon signed-rank test was used for comparison with baseline. PPD, periodontal pocket depth; BOP, bleeding on probing; IQR, interquartile range; CI, Confidence interval.
