# Supplementary material for: Effect of advanced periodontal self-care in patients with early-stage periodontal diseases on endothelial function: An open-label, randomized controlled trial
Source: PLoS One. 2021 Sep 23;16(9):e0257247. doi: 10.1371/journal.pone.0257247 (PMC8459983; doi:10.1371/journal.pone.0257247)
Supplement: Attachment — Submitted filename: 02 Response to Reviewers.docx [file pone.0257247.s008.docx]

Response to Reviewers

We are grateful to each reviewer for the valuable feedback and critical comments, which have helped us improve our manuscript considerably. As indicated in the responses that follow, we have taken all these comments and suggestions into account in the revised version of our manuscript.

Point-by-point responses to Reviewer #1

Abstract

Comment:

- Remember to provide as much as information as possible here. Plos One accepts 300 words with this section, and you should enrich your information.

- With your conclusions in mind, please revise carefully, and see comments given below.

**Response:**

Based on this comment, we have added the sentences of the background and aim of the study in the revised manuscript (Lines 33–41). The word count of the Abstract is now 295 words.

We have taken all the Reviewer #1's comments and suggestions into account in the revised version of our manuscript.

Intro

Comment:

- Aims and objectives have been satisfyingly elaborated.

**Response:**

Thank you for your positive evaluation.

Comment:

- Please note that this section must not provide what you have done (but, instead, what you were going to do). Phrases like "In an RCT, we determined the effect (...)." or "We attempted to advance (...)." must be carefully revised.

**Response:**

Based on this comment, we have replaced “Therefore, advanced self-care may contribute to improving FMD. In an RCT, we determined the effect of advanced periodontal self-care in patients with early-stage periodontal diseases on endothelial function. FMD was set as the primary outcome, while serum asymmetric dimethylarginine (ADMA; an endogenous NO synthase inhibitor) level was the secondary outcome. Elevated serum ADMA is thought to impair endothelial function and, thus, promote atherosclerosis [20]. We attempted to advance periodontal self-care in patients with early-stage periodontal disease to determine any effect on ACVD-related vascular function markers FMD and ADMA.” in the original manuscript (lines 82–90) with “Therefore, we hypothesized that advanced self-care improves endothelial function. This randomized clinical trial aimed to investigate the effect of advanced self-care on ACVD-related vascular function markers.” in the revised manuscript (Lines 89–91).

Comment:

- Please provide a reasonable null hypothesis. Remember that H0 must be deducible from the foregoing thoughts.

**Response:**

Based on this comment, we have added the following sentence:

“Our null hypothesis was that there was no difference in FMD or serum ADMA level between advanced self-care was compared and standard care in patients with early-stage periodontal disease” (Lines 91–92).

Meths

Comment:

- What is meant when referring to "whole-mouth scaling and root planning"? Performed in one visit? Please clarify.

**Response:**

We would like to mean “whole-mouth scaling in a single visit”. Based on this comment, we have replaced “whole-mouth scaling” in the original manuscript (Line 121) with “same-day full-mouth scaling” in the revised manuscript (Line 123).

The “same-day full-mouth root planning” was used in the title of a clinical trial report*.

We have deleted “root planning”. No subjects needed to receive root planning because of their low severity of periodontal diseases.

* Apatzidou DA and Kinane DF. Quadrant root planing versus same-day full-mouth root planing. I. Clinical findings. J Clin Periodontol. 2004; 31: 132-140. doi: 10.1111/j.0303-6979.2004.00461.x.

Comment:

- Please refer to the CONSORT statement with your full text.

**Response:**

We have reviewed overall manuscript to adjust it according to the CONSORT statement and replaced with “S1 CONSORT NPT Extension Checklist”.

Comment:

- Additionally, please stick to the SQUIRE guidelines. You are reporting new knowledge about how to improve healthcare, right?

**Response:**

Thank you for your advice. However, we are not focusing the improvement of the quality of healthcare. The effectiveness of oral care has not been established on the improvement of endothelial function. Therefore, we must clarify the effectiveness of oral care prior to the evaluation of quality improvement in this study. The reason for setting the standard care group as the control group is due to ethical decision. Given that all the subjects were diagnosed with early-stage periodontal diseases, they have a right to receive standard care. We would like you to understand that we are not reporting the improvement of the quality of healthcare.

Comment:

- This was an intention-to-treat study, right? Please refer to the respective analysis.

**Response:**

Based on this comment, we have added revised S1 Table, S2 Table, and S3 Figure showing per-protocol analysis on vascular function and periodontal status in the revised manuscript, respectively.

Comment:

- "ultrasonic scaler", "hand instruments", "dental plaque-disclosing agent", "polishing paste", "rotating rubber cup", "rotating brush", and so on: Please note with ALL materials (including chemicals) and methodologies (including statistical software), please use general names with your text, followed by (brand name; manufacturer, city, STATE (abbreviated, if US), country) in parentheses. Stick to semicolon. Revise thoroughly throughout your text.

- Remember that reproducibility is the cornerstone of scientific advancement. Outputs like exact methodology protocols empower researchers to go one step further in contextualizing their work to ensure it remains replicable. This section has not been satisfying elaborated.

**Response:**

Based on this comment, we have reviewed overall the manuscript and added the required information about the equipment.

“ultrasonic scaler (Varios970; Nakanishi, Tochigi, Japan)” (Line 129).

“hand instruments (FP scaler; Feed, Yokohama, Japan)” (Line 129).

“dental floss （Reach No-waxed; Johnson & Johnson, Tokyo, Japan)” (Line 130)

“dental plaque-disclosing agent (Merssage PC Pellet Blue; Shofu, Kyoto, Japan)” (Line 131).

“polishing paste (PTC Paste Fine/PTC Paste Regular; GC, Tokyo, Japan)” (Line 133)

“rotating rubber cup (FP rubber; Feed, Yokohama, Japan)” (Lines 133–134).

“rotating brush (FP profy brush; Feed, Yokohama, Japan)” (Line 134).

“dental plaster (New Plastone II; GC, Tokyo, Japan)” (Line 158).

“vacuum-forming machine (Biostar; Scheu Dental, Iserlohn, Germany:)” (Lines 161–162).

Comment:

- Do not use legal terms with your text. Delete "Corporation", "Corp.", "Co. Ltd.", "Co.", "Ltd.", "Inc.",

**Response:**

Based on this comment, we have gone through the entire manuscript and deleted the legal terms.

Comment:

- "A single examiner collected (...)." Please provide initials in parentheses.

**Response:**

Based on this comment, we have added the “(A.O.)” in the revised manuscript (Line 195).

Comment:

- "−30°C" must read "-30 °C". Use minus/hyphen instead of dash. The unit is "°C", and must be separated from the number.

**Response:**

Based on this comment, we have replaced “−30°C” in the original manuscript (Line 201) with “-30 ℃” in the revised manuscript (Line 218).

Comment:

- Report exact p-values for all values greater than or equal to 0.001 (note the 3-digit basis). P-values less than 0.001 may be expressed as p < 0.001. Remember to use lowercase letter p. See Authors' Guidelines, and revise thoroughly.

**Response:**

Based on this comment, we have reviewed overall the manuscript and revised the appropriate p-values.

Results

Comment:

- Again, double check and revise p values.

**Response:**

Based on this comment, we have reviewed overall the manuscript and revised the appropriate p-values.

Disc

Comment:

- Stick to H0 when staring this section. Remember that H0 can be rejected or not rejected.

**Response:**

Based on this comment, we have added the following sentence:

Advanced periodontal self-care for three months did not significantly improve FMD as compared to standard care (Lines 303–304).

Comment:

- This section would seem perfectible, please add more discursive thoughts on your outcome.

**Response:**

Thank you for your positive evaluation. Based on this comment, we have added the following sentences in the revised manuscript:

Reduced FMD is associated with the risk of ACVD and improves with risk-reduction therapy. Consequently, endothelial function has been defined as an “excellent barometer” of vascular health. FMD assessing vascular endothelial function might be ideal for exploring factors associated with the improvement of vascular health (Lines 308–311).

Concl

Comment:

- You aimed to "determine the effect of advanced periodontal self-care in patients with early-stage periodontal diseases on endothelial function". Additionally, you "attempted to advance periodontal self-care in patients with early-stage periodontal disease to determine any effect on ACVD-related vascular function markers FMD and ADMA." Hence, please adapt your conclusion ("Advanced periodontal self-care, in addition to standard care, did not result in better vascular function in patients with mild-to-moderate periodontal diseases in this trial.") to your aims. Do not simply repeat your results, but provide a reasonable extension of your outcome.

**Response:**

Based on this comment, we have replaced “Advanced periodontal self-care, in addition to standard care, did not result in better vascular function in patients with mild-to-moderate periodontal diseases in this trial” in the original manuscript (Lines 329–330) with “Advanced periodontal self-care for three months did not significantly improve FMD as compared to standard care in patients with early-stage periodontal diseases. Neither care significantly improved FMD despite its effectiveness in improving periodontal status. (Lines 362–364).

Comment:

In total, this submitted draft would seem interesting, is considered easily intelligible, and should be worth following after revision. This paper is ready for external review.

**Response:**

Thank you for your positive evaluation. We hope that our explanations and revisions are satisfactory.

Point-by-point responses to Reviewer #2

In the manuscript entitled: “Effect of advanced periodontal self-care in patients with early-stage periodontal diseases on endothelial function: An open-label, randomized controlled trial”, the authors identified the effects of advanced periodontal selfcare on endothelial function in patients with early-stage periodontal disease. The study was designed as a parallel group, 3-month follow-up, open-label, randomized controlled trial for evaluating the effectiveness of periodontal care against atherosclerotic cardiovascular disease.

The authors found that both groups demonstrated improved periodontal status. No significant improvements in FMD were observed in the control or test group. No significant changes in serum ADMA levels were observed. No significant between-group differences were found in FMD or serum ADMA levels.

The authors concluded that periodontal care for a 3-month duration did not provide better endothelial function in patients with early-stage periodontal diseases.

Major comments:

In general, the idea and innovation of this study, regards analysis of eriodontal self-care in patients with early-stage periodontal diseases on endothelial function is interesting, because the role of these factors in dentistry are validated but further studies on this topic could be an innovative issue in this field could be open a creative matter of debate in literature by adding new information. Moreover, there are few reports in the literature that studied this interesting topic with this kind of study design.

The study was well conducted by the authors; However, there are some concerns to revise that are described below.

Comment:

The introduction section resumes the existing knowledge regarding the important factor linked with periodontal inflammation. However, as the importance of the topic, the reviewer strongly recommends, before a further re-evaluation of the manuscript, to update the literature through read, discuss and must cites in the references with great attention all of those recent interesting articles, that helps the authors to better introduce and discuss the role of periodontitis and related biomarkers as cause of implant failure (Galectin, NLRP3): 1) Isola G, Polizzi A, Alibrandi A, Williams RC, Lo Giudice A. Analysis of galectin-3 levels as a source of coronary heart disease risk during periodontitis. J Periodontal Res. 2021 Jun;56(3):597-605. doi: 10.1111/jre.12860. 2) Isola G, Polizzi A, Santonocito S, Alibrandi A, Williams RC. Periodontitis activates the NLRP3 inflammasome in serum and saliva. J Periodontol. 2021 May 19. doi: 10.1002/JPER.21-0049. 3) Isola G, Lo Giudice A, Polizzi A, Alibrandi A, Murabito P, Indelicato F. Identification of the different salivary Interleukin-6 profiles in patients with periodontitis: A cross-sectional study. Arch Oral Biol. 2021 Feb;122:104997. doi: 10.1016/j.archoralbio.2020.104997.

**Response:**

Thank you for your critical advice. As mentioned by Reviewer #2, periodontal inflammation may induce atherosclerotic cardiovascular disease through not only bacteremia but also signal transduction involved in inflammatory mediators.

Based on this comment, we have added the following sentence with citations of all the introduced references in the revised manuscript:

“Furthermore, it has also been suggested that inflammatory mediators attributable to periodontal diseases are associated with ACVD” (Lines 61 –62).

Comment:

The authors should be better specified, at the end of the introduction section, the rational of the study and the aim of the study.

**Response:**

Based on this comment, we have added the following sentence in the revised manuscript:

Therefore, we hypothesized that advanced self-care improves endothelial function. The aim of this randomized clinical trial was to investigate the effect of advanced self-care on ACVD-related vascular function markers.” (Lines 89–91).

Comment:

In the material and methods section, should better clarify randomization* and periodontal examination**.

**Response:**

*Based on this comment, we have added the following sentences in the revised manuscript:

“All the researchers confirmed that no one could identify the number” (Lines 184–185).

“After eligibility assessment, each patient selected as a study subject made an appointment for random allocation at their convenience.” (Lines 188–189).

“Balloting was done on a first-come, first-served basis. The allocated Arabic number was particular for each subject, and the number was never used again” (Lines 190–192).

** Based on this comment, we have replaced “A single examiner collected the following clinical data from six sites around each tooth” in the original manuscript (Line 182) with “A single examiner (A.O.) collected the following clinical data from six sites (mesio-buccal, mid-buccal, disto-buccal, mesio-lingual, mid- lingual, and disto-lingual) around each tooth using a color-coded periodontal probe (PO-9; Nippon Shiken, Tokyo, Japan) in the revised manuscript (Lines 195–197).

Comment:

Moreover, please more specific the clinicians involved in the different stages of the study.

**Response:**

Based on this comment, we have provided initials of each clinician or qualified medical personnel in parentheses in each of the different stages in the revised manuscript:

Authors: R.O., M.S., H.A., H.U., A.I., Non-authors: refer to Acknowledgments; T.K., E.S., N.A., Y.U., M.E., J.T. (Lines 125–126),

T.M. (Line 136, 146)

A.O. (Line 195, 214).

Comment:

The discussion section appears well organized with the relevant paper that support the conclusions, even if the authors should better discuss the relationship between periodontitis and endothelial dysfunction.

**Response:**

Based on this comment, we have added the following sentence with a citation concerning ACVD through inflammatory mediators in the revised manuscript:

“In addition, production of inflammatory mediators attributable to periodontal diseases might be associated with ACVD (Lines 306–308).

Comment:

The conclusion should reinforce in light of the discussions.

**Response:**

Based on this comment, we have replaced “Advanced periodontal self-care, in addition to standard care, did not result in better vascular function in patients with mild-to-moderate periodontal diseases in this trial” in the original manuscript (Lines 329–330) with “Advanced periodontal self-care for three months did not significantly improve FMD as compared to standard care in patients with early-stage periodontal diseases. Neither care significantly improved FMD despite its effectiveness in improving the periodontal status.” in the revised manuscript (Lines 362–364).

Comment:

In conclusion, I am sure that the authors are fine clinicians who achieve very nice results with their adopted protocol. However, this study, in my view does not in its current form satisfy a very high scientific requirement for publication in this journal and requests a revision before a further re-evaluation of the manuscript.

**Response:**

We have addressed all the comments by Reviewer #2. We hope that our explanations and revisions are satisfactory.

Minor Comments

Abstract

Comment:

- Better formulate the Abstract section by better describing the aim of the study

**Response:**

Based on this comment, we have added the following sentence in the Abstract:

This randomized clinical trial aimed to investigate the effect of advanced self-care on ACVD-related vascular function markers flow-mediated brachial artery dilatation (FMD) and serum asymmetric dimethylarginine (ADMA) levels in patients with early-stage periodontal disease (lines 37–41)

Introduction

Comment:

- Please refer to major comments

**Response:**

Based on the major comment above, we have added the following sentence with citation in the revised manuscript:

Furthermore, it has also been suggested that inflammatory mediators attributable to periodontal diseases are associated with ACVD (Lines 61–62).

Discussion

Comment:

- Please add a specific sentence that clarifies the results obtained in the first part of the discussion

**Response:**

Based on this comment, we have added the following sentences in the revised manuscript:

Advanced periodontal self-care for three months did not significantly improve FMD as compared to standard care. (Lines 303–304).

Comment:

- Page 16 last paragraph: Please reorganize this paragraph that is not clear

**Response:**

Based on this comment, we have added “than in patients with early-stage periodontal diseases” in the revised manuscript (Line 324).

Point-by-point responses to Reviewer #3

Comment:

1. Sample size calculation needs more details. What is the mean (SD) of each group at each time point? What test was used?

**Response:**

We did not have enough data prior to the trial. Therefore, we referred to previous similar trials (Tonetti MS, et al. Treatment of periodontitis and endothelial function. N Engl J Med. 2007; 356: 911-920.) as described in the original manuscript (Lines 164–167) and the revised manuscript (Lines 173–176). The authors set: effect size; 1%, standard deviation of the mean difference: 1.67%.

We have added the following sentence “We employed a priori power analysis for sample size calculation” in the revised manuscript (Line 173).

Comment:

2. Linear mixed models (repeated ANOVA) may be considered for analyzing the outcomes. If data/model residuals follow normal distributions, it is more powerful to use parametric methods.

**Response:**

Thank you for your advice. There are two time points in this trial (baseline and endpoint). Therefore, the paired *t*-test is used for comparison between baseline and endpoint. Repeated ANOVA should be used in cases where there are more than three time points.

Comment:

3. Results:

a. Table 1: add p values. Specify methods used for comparison in the footnote.

**Response:**

We show the baseline comparison in Table 1. As described in “How to Report Statistics in Medicine (2^nd^ ed.) (Thomas A. Lang, Michelle Secic. American College of Physicians Philadelphia 2006, pp 207-208^1^), the p-values for baseline comparisons would not be necessary in randomized trials. Therefore, we did not report the p-values for baseline comparison. We have removed the description of the baseline comparison on vascular function from the original manuscript (Lines 256–257).

^1^ It is not necessary to report the p*-*values for baseline comparisons in randomized trials. In such trials, any differences between groups in baseline variables will be the result of chance because participants were assigned to groups at random. Baseline comparisons do need to be made, however, to identify any statistical imbalances that may need to be adjusted for in the final multivariable model. If p-values are reported for baseline comparisons in a randomized trial, they should be interpreted only as measure of the strength of the imbalance between the groups, not as evidence of bias.

Comment:

b. Periodontal condition: move this section after “Vascular function” as they are secondary outcomes*. Show median (IQR) for each group at each time point, not just improvement**. Add all *p* *value*s for within-group comparisons and between-group comparisons**.

**Response:**

* Based on this comment, the description of “Vascular function” is followed by that of “Periodontal condition” in the revised manuscript.

** Based on these comments, we have replaced Table 2 and S1 Table in the original manuscript with revised Table 3 (ITT) and S2 Table (per-protocol analysis) in the revised manuscript, respectively. The revised tables show the median (IQR) for each group at endpoint with p-values in addition to the mean improvement (95% CI). Baseline data have already been described in Table 1. The revised S2 Table includes baseline data because of no descriptions on baseline for per-protocol analysis in the manuscript. We have removed statistical results from the text to avoid duplicate descriptions.

Comment:

c. Vascular function: add a table with detailed statistics, e.g. mean (SD) /median (IQR) for each group at each time point. Add all p values for within-group comparisons and between-group comparisons. Need to perform a consistency analysis for ITT and per-protocol also.

**Response:**

Based on this comment, we have added revised Table 2 (ITT) and S1 Table (per-protocol analysis) in the revised manuscript. The revised tables show the mean (SD) or median (IQR) for each group at endpoint with p-values in addition to the mean improvement (95% CI). Baseline data have already been described in Table 1. The revised S1 Table includes baseline data because of the absence of descriptions on baseline for per-protocol analysis in the manuscript. We have removed statistical results from the text except mean improvement between group to avoid duplicate descriptions. We have deleted Fig. 2 in the original manuscript to avoid duplicate descriptions. However, figures are generally preferred to tables for presentations of comparisons^2^. Therefore, we have shown the revised figures as supporting information (S2 Fig and S3 Fig).

^2^ How to Report Statistics in Medicine (2^nd^ ed.). Thomas A. Lang, Michelle Secic. American College of Physicians Philadelphia 2006, pp 328

“We recommend that every effort be made to present comparisons in figures rather than in tables, even when the amount of data is small.”

Comment:

d. Figure 2: using boxplot for both outcomes. Specify clearly which methods were used for generating the p values.

**Response:**

Based on this comment, we have replaced the original Figure 2 with revised S2 Fig, indicating the results with boxplots. The boxplots are presented as supporting information to avoid duplicate descriptions. We have also adjusted the corresponding captions. Statistical procedures were described in the captions.

Comment:

e. Line 275 is not a complete sentence.

**Response:**

Line 275 contains definitions of abbreviations and a part of Figure captions. We have undone the line break in the revised manuscript.

CONSORT checklist:
1) Outcomes are clearly defined.

Complete

2) Sample size needs more details for mean (SD) in each group, not mean difference (SD). What test was used?

**Response:**

As described above, we did not have enough data prior trial. Therefore, we referred to previous similar trials (Tonetti MS, et al. Treatment of periodontitis and endothelial function. N Engl J Med. 2007; 356: 911-920.) as described in the original (Lines 164–167) and revised (Lines 173–176) manuscripts. The authors set: effect size; 1%, standard deviation of the mean difference: 1.67%.

We have added the following sentence in the revised manuscript “We used a priori power analysis for sample size calculation” (Line 173).

3) Method used to generate random allocation sequence was not mentioned.

**Response:**

We have added the following sentences to clarify the random allocation sequence in the revised manuscript:

“All the researchers confirmed that no one could identify the number (Lines 184–185).

“After eligibility assessment, each patient selected as a study subject made an appointment for random allocation at their convenience.” (Lines 188–189).

“Balloting was done on a first-come, first-served basis. The allocated Arabic number was particular for each subject, and the number was never used again.” (Lines 190–192).

4) Allocation concealment was clearly described.

Complete

5) Blinding is not available.

**Response:**

This trial is open-labeled.

6) Details of outcomes and estimation were not provided*. Need means (SD) for each group at each time point and mean different (with 95% CI)**. A table is necessary**. Effect size was not provided either*.

**Response:**

* We have added the following sentences to the revised manuscript:

“An improvement in FMD of at least 1% difference was not achieved between the groups.” (Line 261).

We set the effect size at 1% difference in FMD as described in the original (Line 165) and revised (Line 174) manuscripts.

** We have added revised Table 2 (ITT) and S1 Table (per-protocol analysis) showing the mean ± SD/median (IQR) for each group at endpoint and mean difference (with 95% CI) in the revised manuscript.

7) Important harms or unintended effects were not discussed.

**Response:**

We have added the following sentence:

Harms

No study-related serious adverse events occurred in any of the study participants. Nonsurgical periodontal treatment is basically low-risk. None of the participants required any dental therapy during the study. (Lines 297–300).

8) The registry and the registration number were reported.

Complete

9) The trial protocol is attached.

Complete

10) Sources of funding was described.

Complete
