# Supplementary material for: Effect of advanced periodontal self-care in patients with early-stage periodontal diseases on endothelial function: An open-label, randomized controlled trial
Source: PLoS One. 2021 Sep 23;16(9):e0257247. doi: 10.1371/journal.pone.0257247 (PMC8459983; doi:10.1371/journal.pone.0257247)
Supplement: Attachment — Submitted filename: 04 Response to Reviewers.docx [file pone.0257247.s009.docx]

Response to Reviewers

Point-by-point responses to Reviewer #1

Comment:

No doubt, this revised draft has been considerably increased. However, some minor aspects would still seem in need of further revisions, please see below.

**Response:**

We are grateful to Reviewer #1 for the second review and for providing advice. As indicated in the responses that follow, we have taken all these comments and suggestions into account in the revised version of our manuscript.

Comment:

- With reference to your Abstract section, do not provide a shortened literature review, please. Instead, please focus more intensely on your outcome.

**Response:**

Based on this comment, we have added the details of between-group differences in FMD and serum ADMA levels, and results of periodontal status in the Abstract.

For example,

“(mean difference, −0.2%; 95% CI, −1.4–0.9; *p* = 0.708)” (Line 46).

“(mean difference, 0.01 nmol/L; 95% CI, −0.00–0.03; *p* = 0.122)” (Lines 46–47).

“Significant improvements in the average probing pocket depth were observed in the control and test groups. The bleeding on probing score in the test group was significantly reduced, while that in the control group was reduced, although not significantly” (Lines 47–50).

Comment:

-"The immune response following persistent bacteremia from periodontal lesions may lead to ACVD [3]. Furthermore, it has also been suggested that inflammatory mediators attributable to periodontal diseases are associated with ACVD [4-6]." Consequently, "bacteremia-induced ACVD routinely occurs even in the early stage of periodontal disease [2,19]". Undoubtedly, this would seem right. However, this would lead to the idea of a thorough eradication of all biofilms deemed responsible for infection, persistent bacteremia, and presence of inflammatory mediators (due to the biofilm). This refers to your "same-day full-mouth scaling, an ultrasonic scaler, along with hand instruments, and dental floss, and polishing paste with rotating rubber cup/brush", which is not deemed sufficient (even if widely established). Please refer to <https://pubmed.ncbi.nlm.nih.gov/34269042/>; there you will see that "(not only supragingival) biofilm removal by means of air polishing combined with low-abrasive erythritol has been shown to be more efficacious than the traditional polishing method". Thus, your applied methodology should be discussed more thoroughly, in particular since "thorough biofilm removal clearly results in delayed plaque regrowth rates", thus potentially showing a clear influence on inflammatory mediators and ACVD, in particular in combination with your "extra intervention in addition to standard self-care". Again, go to <https://doi.org/10.3290/j.qi.b1763661>, and discuss, both with regard to general effects and with regard to possible limitations.

**Response:**

Thank you for bringing this recent paper to our attention. The paper shows that “polishing combined with low-abrasive erythritol” is more efficacious than “air polishing or rubber cups with prophylaxis paste” for biofilm removal and delayed biofilm regrowth rates within 24 hours. As mentioned by Reviewer #2, the outcomes may be affected by the method employed for biofilm removal.

Based on this comment, we have added the following sentence to the revised manuscript with an appropriate citation:

“The main contents of standard care at the clinic are biofilm removal. Employment of more effective methods for biofilm removal may affect the improving FMD through delayed biofilm regrowth rates [28].” (Lines 325–327).

Comment:

- "We considered a 70% implementation rate as successful advanced periodontal self-care." Do you mean "successfully advanced periodontal self-care"?

**Response:**

Yes, we do. We decided that a “70% implementation rate represented successful advanced periodontal self-care” before the trial began. The decision was made in consideration of the burden on the subject and our empirical tolerance level for "successfully advanced periodontal self-care.” Those subjects with a below “70% implementation rate” were excluded from per-protocol analysis.

Comment:

- "(1–29 for smokers and 1–81 for nonsmokers)" should read "(1 to 29 for smokers and 1 to 81 for nonsmokers)".

**Response:**

Based on this comment, we have replaced “1–29 for smokers and 1–81 for nonsmokers” with “1 to 29 for smokers and 1 to 81 for nonsmokers” in the revised manuscript (Line 182).

Comment:

- "All the researchers confirmed that no one could identify the number." This would not seem clear, please clarify.

**Response:**

Based on this comment, we have added “from the outside of each sealed envelope” (Line 183).

Comment:

- "(Unexef18G, UNEX, Nagoya, Japan)" must read "(Unexef18G; UNEX, Nagoya, Japan)". Double check thoroughly.

**Response:**

Based on this comment, we have replaced “(Unexef18G, UNEX, Nagoya, Japan)” with “Unexef18G; UNEX, Nagoya, Japan” in the revised manuscript (Lines 203–204).

Comment:

- With your results section, please add exact p values. (See, for example, "Significant improvements in the average PPD were observed in the control and test groups. The BOP score in the test group was significantly reduced, while that in the control group was reduced, although not significantly.").

**Response:**

We have added a revised Table 3 and S2 Table based on the other reviewer’s comment. We have shown the exact *p* values in the Tables. We have removed statistical results from the text to avoid duplicate descriptions in the revised manuscript.

Comment:

- Again, with the first paragraph of your Disc section, please refer explicitly to H0, to provide a clear answer. "The findings were similar in the intention-to-treat and the per-protocol analysis." should read ""The findings were similar in the intention-to-treat and the per-protocol analysis; thus H0 was not rejected.

**Response:**

Thank you for your observation. Based on this comment, we have added the following sentence:

“Therefore, our null hypothesis ‘there was no difference in FMD between advanced self-care and standard care in patients with early-stage periodontal disease’ was not rejected.” (Lines 304–305).

Comment:

- "ADMA is a naturally occurring endogenous inhibitor of NO synthase." Reference(s) missing.

**Response:**

Based on this comment, we have added the following three references:

29. Najbauer J, Johnson BA, Young AL, Aswad DW. Peptides with sequences similar to glycine, arginine-rich motifs in proteins interacting with RNA are efficiently recognized by methyltransferase(s) modifying arginine in numerous proteins. J Biol Chem. 1993; 268(14): 10501-10509. PMID: 7683681.
30. Tang J, Kao PN, Herschman HR. Protein-arginine methyltransferase I, the predominant protein-arginine methyltransferase in cells, interacts with and is regulated by interleukin enhancer-binding factor 3. J Biol Chem. 2000; 275(26): 19866-19876. https://doi.org/10.1074/jbc.M000023200 PMID: 10749851.
31. MacAllister RJ, Fickling SA, Whitley GS, Vallance P. Metabolism of methylarginines by human vasculature; implications for the regulation of nitric oxide synthesis. Br J Pharmacol. 1994; 112(1): 43-48. https://doi.org/10.1111/j.1476-5381.1994.tb13026.x PMID: 7518309.

Comment:

- Same with "It reduces NO production; consequently, it can lead to endothelial dysfunction and cardiovascular events." Please revise carefully, and double check your whole draft, to support statements.

**Response:**

Based on this comment, we have added the following four references:

24. Sibal L, Agarwal SC, Home PD, Boger RH. The role of asymmetric dimethylarginine (ADMA) in endothelial dysfunction and cardiovascular disease. Curr Cardiol Rev. 2010; 6(2): 82-90. https://doi.org/10.2174/157340310791162659 PMID: 21532773.
32. Achan V, Broadhead M, Malaki M, Whitley G, Leiper J, MacAllister R, et al. Asymmetric dimethylarginine causes hypertension and cardiac dysfunction in humans and is actively metabolized by dimethylarginine dimethylaminohydrolase. Arterioscler Thromb Vasc Biol. 2003; 23(8): 1455-1459. https://doi.org/10.1161/01.ATV.0000081742.92006.59 PMID: 12805079.
33. Kielstein JT, Impraim B, Simmel S, Bode-Böger SM, Tsikas D, Frölich JC, et al. Cardiovascular effects of systemic NO synthase inhibition with asymmetric dimethylarginine in humans. Circulation. 2004; 109(2): 172-177. https://doi.org/10.1161/01.CIR.0000105764.22626.B1 PMID: 14662708.
34. Fliser D. Asymmetric dimethylarginine (ADMA): the silent transition from an ‘uraemic toxin’ to a global cardiovascular risk molecule. Eur J Clin Invest. 2005; 35(2): 71-79. https://doi.org/10.1111/j.1365-2362.2005.01457.x PMID: 15667575.

Comment:

- "Further investigations may be required in healthy subjects." To study what kind of effects? Please clarify.

**Response:**

Based on this comment, we have replaced “Further investigations may be required in healthy subjects” with “The reason for the discrepancy between the trials may be the underlying factors characterizing the study population which may be affecting FMD and/or plasma ADMA levels. Further investigations may be required to identify the factors in healthy subjects.” (Lines 336–339).

Comment:

- "A longer period might be required to alter endothelial function in patients with mild-to-moderate periodontal diseases." Please provide reasons why you think that prolonged follow-up periods would change the outcome.

**Response:**

When a certain treatment does not give positive results in a short span of time, continuation of the treatment is one of the common options available to medical practitioners. A longer period of care may increase the effectiveness of treatment for persistent bacteremia from periodontal lesions in patients with mild-to-moderate periodontal diseases.

Based on this comment, we have added “, which enables further improvement in bacteremia,” (Line 364).

Comment:

- Regarding the reference list, again, revise for uniform formatting (see Guidelines). Format must be "Bürgers R, Eidt A, Frankenberger R, Rosentritt M, Schweikl H, Handel G, et al. The anti-adherence activity and bactericidal effect of microparticulate silver additives in composite resin materials. Arch Oral Biol. 2009; 54(6): 595-601. <https://doi.org/10.1016/j.archoralbio.2009.03.004> PMID: 19375069". Please revise for spacebar use to separate year and volume. Provide issue numbers (if available). Revise for correct doi numbers, and provide PMID numbers.

**Response:**

Based on this comment, we have reviewed all the references and adjusted them according to the Guidelines.

Comment:

Again, this study would seem worth following after revision. Compliments to the authors!

**Response:**

Thank you for the encouragement. We hope that our explanations and revisions are satisfactory.
